# Transcriptomic and metabolomic changes in postharvest sugarbeet roots reveal widespread metabolic changes in storage and identify genes potentially responsible for respiratory sucrose loss

**DOI:** 10.3389/fpls.2024.1320705

**Published:** 2024-01-30

**Authors:** Karen K. Fugate, John D. Eide, Abbas M. Lafta, Muhammad Massub Tehseen, Chenggen Chu, Mohamed F. R. Khan, Fernando L. Finger

**Affiliations:** ^1^ Edward T. Schafer Agricultural Research Center, U.S. Department of Agriculture, Agricultural Research Service, Fargo, ND, United States; ^2^ Department of Plant Pathology, North Dakota State University, Fargo, ND, United States; ^3^ Department of Plant Sciences, North Dakota State University, Fargo, ND, United States; ^4^ University of Minnesota Extension Service, St. Paul, MN, United States; ^5^ Departamento de Agronomia, Universidade Federal de Viçosa, Viçosa, Brazil

**Keywords:** *Beta vulgaris*, bidirectional sugar transporter, glycolysis, pyruvate kinase, respiration, SWEET gene

## Abstract

Endogenous metabolism is primarily responsible for losses in sucrose content and processing quality in postharvest sugarbeet roots. The genes responsible for this metabolism and the transcriptional changes that regulate it, however, are largely unknown. To identify genes and metabolic pathways that participate in postharvest sugarbeet root metabolism and the transcriptional changes that contribute to their regulation, transcriptomic and metabolomic profiles were generated for sugarbeet roots at harvest and after 12, 40 and 120 d storage at 5 and 12°C and gene expression and metabolite concentration changes related to storage duration or temperature were identified. During storage, 8656 genes, or 34% of all expressed genes, and 225 metabolites, equivalent to 59% of detected metabolites, were altered in expression or concentration, indicating extensive transcriptional and metabolic changes in stored roots. These genes and metabolites contributed to a wide range of cellular and molecular functions, with carbohydrate metabolism being the function to which the greatest number of genes and metabolites classified. Because respiration has a central role in postharvest metabolism and is largely responsible for sucrose loss in sugarbeet roots, genes and metabolites involved in and correlated to respiration were identified. Seventy-five genes participating in respiration were differentially expressed during storage, including two bidirectional sugar transporter SWEET17 genes that highly correlated with respiration rate. Weighted gene co-expression network analysis identified 1896 additional genes that positively correlated with respiration rate and predicted a pyruvate kinase gene to be a central regulator or biomarker for respiration rate. Overall, these results reveal the extensive and diverse physiological and metabolic changes that occur in stored sugarbeet roots and identify genes with potential roles as regulators or biomarkers for respiratory sucrose loss.

## 1 Introduction

Following harvest, most of the sugarbeet (*Beta vulgaris* L.) crop is stored in outdoor piles or ventilated sheds as roots await processing into sugar. Piles commonly contain 100 to 1000 tons (t) of roots in European countries and up to and exceeding 100,000 t of roots in the U.S., and are cooled, passively or via ventilation systems, using cold ambient winter air (Campbell and Klotz, 2006; Bernhardson, 2009; English, 2020). Piles are optimally maintained at temperatures of 2 to 8°C (Huijbregts et al., 2013). However, higher pile temperatures are not uncommon due to the sheer mass of roots that must be cooled following harvest, the reliance on weather conditions that are often insufficiently cold, and the limited ability of pile ventilation to fully dissipate heat released by the metabolism of roots and postharvest pathogens (Kays and Paull, 2004; Campbell and Klotz, 2006);. Roots are stored for up to 60 d in Europe (Huijbregts et al., 2013). In the U.S., storage lasts for as long as 280 d, with piles maintained at cold, nonfreezing temperatures in the first 100 to 140 d of storage then frozen during the coldest days of winter and maintained frozen for the duration of storage (Bernhardson, 2009).

Sugarbeet root sucrose content and processing quality decline during storage, reducing the quantity of sugar that can be recovered during processing and increasing production costs. Losses of 3 to 10% of the sucrose present at harvest are reported for roots stored up to 100 d under favorable temperature conditions, but these can escalate to 50% or more if optimal pile temperatures are not obtained or maintained (Kenter and Hoffmann, 2009; Beaudry et al., 2011; English, 2020). Processing quality deteriorates due to the formation and accumulation of carbohydrate impurities, such as glucose, fructose and raffinose, and cell wall modifications, that combined with root dehydration, soften roots (Vukov and Hangyál, 1985; Dutton and Huijbregts, 2006). Non-sucrose carbohydrates reduce recoverable sucrose yield and increase processing time and cost; softening impedes root slicing and increases the solubilization of cell wall components such as pectins, complicating and slowing sugar recovery in the factory (Dutton and Huijbregts, 2006).

Sugarbeet root metabolism is largely responsible for reductions in sucrose content and processing quality during storage. As defoliation at harvest separates roots from their pre-harvest source of photosynthate, sugarbeet roots rely on stored sucrose to fuel their metabolism until they are frozen for long-term storage or processed in the factory. Central to postharvest metabolism is respiration, a complex, multi-enzyme process that converts storage compounds into metabolic energy to maintain cellular functions, but causes 60 to 80% of sugarbeet root sucrose loss during storage (Wyse and Dexter, 1971b; Vukov and Hangyál, 1985; Kays and Paull, 2004). Extensive injuries sustained by roots from harvest and piling activities additionally activate secondary metabolism for lignin and suberin biosynthesis and the induction of plant immunity to limit dehydration and pathogen infection at wound sites (Fugate et al., 2016; Fugate et al., 2023a). Adaptation to the increasingly cold and often dehydrating conditions of the storage environment require further metabolic changes, including altering lipid metabolism to retain membrane fluidity, enhancing biosynthesis of compatible solutes, such as raffinose, proline and betaine, and activating reactive oxygen species defense mechanisms (Haagenson et al., 2008; Yadav, 2010; Finger et al., 2021). Other metabolic changes known to occur in stored sugarbeet roots include alterations in pectolytic and other cell wall modifying enzyme activities and catabolism and interconversions of amino acids (Wyse and Dexter, 1971a; Vukov and Hangyál, 1985; Tungland et al., 1998; Gippert et al., 2022).

Current knowledge of the biochemical and molecular changes that are responsible for postharvest sugarbeet root metabolism is surprisingly limited and insufficient for understanding sucrose loss and quality deterioration during storage. While information is available on storage-related changes in enzyme activities that participate in sucrose degradation, carbohydrate impurity formation, wound-healing and root softening pathways, this information is generally available for only selected enzyme activities in these metabolic pathways (Vukov and Hangyál, 1985; Klotz and Finger, 2004; Haagenson et al., 2008; Fugate et al., 2016; Megguer et al., 2017). The genes responsible for postharvest metabolism are largely unknown, with only those involved in sucrolytic reactions identified and characterized (Rosenkranz et al., 2001; Klotz and Haagenson, 2008). Recently, transcriptomic differences for sugarbeet varieties with putative differences in their ability to retain sucrose and accumulate invert sugars (i.e., glucose and fructose) during storage were described, providing the first extensive description of genetic changes occurring during storage (Madritsch et al., 2020). However, since storage rots developed, to varying degrees, on all varieties in this study, the genetic differences noted by the authors may describe differences in innate immunity or molecular responses to storage rots and have limited relevance for understanding molecular changes in healthy roots.

To gain a better understanding of molecular changes involved in the endogenous metabolism that causes sucrose and root quality loss during storage, an analysis was made of the transcriptomic and metabolomic changes occurring in sugarbeet roots stored at 5 and 12°C after 12, 40 or 120 d. Storage temperatures were chosen as representative of favorable (5°C) and unfavorable (12°C) temperature conditions, while storage durations allowed the identification of molecular changes occurring in sugarbeet roots during short-term (12 d) and long-term storage for unfrozen roots in Europe (40 d) and the U.S (120 d). Because of the central importance of respiration to postharvest sucrose losses, an in-depth analysis of the changes in genes and metabolites that participate and correlate to root respiration rate during storage was also made. The purpose of this analysis was not only to elucidate the genes and metabolites that participate in this process and characterize their responses to storage temperature and duration, but also to identify genes that may regulate or function as biomarkers for sugarbeet storage respiration rate.

## 2 Materials and methods

### 2.1 Plant material and treatments

Sugarbeet plants (variety VDH66156, SESVanderHave, Tienen, Belgium) were grown from seed in a greenhouse in 15 L pots with 16 h light/8 h dark periods as previously described (Megguer et al., 2017). Eighteen weeks after planting, taproots from 56 plants were harvested. All leaf and petiole materials were excised from roots with a knife, and roots were gently handwashed and allowed to dry. Tissue samples were obtained from eight randomly selected roots on the day of harvest by excising a longitudinal section that was representative of the entire root from each of the eight roots. Tissue samples were rapidly frozen in liquid nitrogen, lyophilized, ground to a fine powder, and stored at -80°C prior to use. The remaining 48 roots were randomly divided into two groups of 24 roots. Each group was stored in an independent chamber of a two-chamber Conviron (Winnipeg, MB, Canada) E7/2 growth chamber unit, with one chamber operating at 5°C, the other chamber operating at 12°C, and both chambers set to 95% relative humidity. Eight roots were randomly removed from each chamber after 12, 40, and 120 d in storage. At each time point, the respiration rates of each of the eight individual roots per temperature treatment were determined as described below, and a tissue sample from each root was collected as described above. For all analyses, individual roots served as replicates with eight replicate roots per time point/storage temperature combination.

### 2.2 Respiration rate determination

Respiration rate was quantified by infrared gas analysis using an open system and expressed as the CO_2_ evolved from each root as a function of root weight (Haagenson et al., 2006). Briefly, individual roots were contained in a 7-L sample chamber to which the gas analyzer from a LI-COR LI-6400 photosynthesis system (Lincoln, NE, USA) was attached. Air flow (1000 μmol s^-1^) was maintained throughout the chamber using the internal pump of the LI-6400 photosynthesis system, and CO_2_ concentration within the chamber was measured when gas concentrations in the chamber equilibrated.

### 2.3 RNA sequencing

Total RNA was isolated from tissue samples collected at harvest, after 12, 40, and 120 d storage at 5°C, and after 12, 40, and 120 d storage at 12°C. RNA was obtained from lyophilized tissue (50 mg) using a RNeasy Plant Mini Kit (QIAGEN, Valencia, CA, USA) with an on-column DNase digestion. RNA concentration was quantified using a ThermoFisher Scientific NanoDrop ND-1000 spectrophotometer (Waltham, MA, USA), and RNA integrity was confirmed by the RIN number generated by an Agilent Technologies 2100 Bioanalyzer (Pal Alto, CA, USA). RNA was enriched in mRNA by oligo dT selection, fragmented, reverse transcribed into cDNA using random primers, amplified by PCR, and sequenced by BGI Americas (Cambridge, MA, USA) using DNBseq technology on a BGISEQ-500 platform. The number of raw reads ranged from 29.5 to 35.0 M per sample, with an average of 29.8 M raw reads per sample.

Raw reads were cleaned by removing reads with adapters, reads with >10% unknown bases and low-quality reads using SOAPnuke ver. 1.5.2 (Chen et al., 2018), leaving an average of 29.3 M clean reads per sample. HISAT2 ver. 2.0.4 (Kim et al., 2019) and Bowtie2 ver. 2.2.5 (Langmead and Salzberg, 2012) were used to map clean reads to the sugarbeet genome (Dohm et al., 2013). Gene expression levels were calculated with RSEM ver. 1.2.12 (Li and Dewey, 2011), and differentially expressed genes with fold change ≥ 2.00 and adjusted Pvalues ≤ 0.05 were detected using DEseq2 (Love et al., 2014). Functional enrichment of DEGs was performed using the phyper function in R.

### 2.4 Metabolomics analysis

Metabolites were extracted from lyophilized tissue and characterized by Metabolon (Durham, NC, USA) using their Metabolon HD4 platform. Extraction was effected by addition of methanol to lyophilized tissue samples, vigorous shaking, and centrifugation to remove insoluble material. Prior to extraction, internal standards were added to monitor recovery rate. Methanol was evaporated from samples and extracted material was redissolved in aqueous solutions containing standards of known concentrations. Extracts were analyzed by reverse phase ultra-performance liquid chromatography tandem mass spectrometry (UPLC-MS/MS) using positive ion electrospray ionization (ESI), UPLC-MS/MS with negative ion ESI analysis, and hydrophilic interaction liquid chromatography (HILIC)/UPLC-MS/MS with negative ion ESI. UPLC-MS/MS was performed on a Waters (Milford, MA, USA) ACQUITY UPLC system and Thermo Scientific (Waltham, MA, USA) Q-Exactive mass spectrometer with heated electrospray ionization source and Orbitrap mass analyzer. Waters UPLC BEH C18 2.1 x 100 mm columns were used for UPLC-MS/MS analyses and a Waters UPLC BEH Amide 2.1 x 150 mm column was used for HILIC/UPLC-MS/MS analyses. Metabolites were identified by comparison of retention time, mass to charge ratio, and MS/MS spectral data to authenticated standards. All data were scaled to the median value for each compound. Significant differences between treatments were determined by ANOVA after natural log transformation of scaled data, with p ≤ 0.05.

### 2.5 Enzyme activity assays

Activity of pyruvate kinase was determined using a previously described enzyme-coupled spectrophotometric assay (Lafta and Fugate, 2009; Megguer et al., 2017). Protein concentrations were determined using Bio-Rad Protein Assay Dye Reagent (Hercules, CA, USA) with bovine serum albumin as a standard.

### 2.6 Data analysis

Heat maps were generated using Next Generation Clustered Heat Map Tool ver. 2.14.4 (NG-CHM, MD Anderson Cancer Center, Houston, TX, USA). Significant differences in respiration rate were determined by ANOVA, with Fisher’s least significant difference (LSD) used to identify treatments that differed from each other (p ≤ 0.05). Significant differences in enzyme activity between data points and with respect to time in storage were determined by ANOVA and regression analysis, with p ≤ 0.05. Fisher’s LSD was used to identify data points that differed significantly from other data points. Principle component, ANOVA, and regression analyses were performed using Minitab Statistical Software, ver 20.4 (State College, PA, USA). Weighted gene co-expression network analysis (WGCNA) was conducted using the WGCNA package, ver 1.51, in R (Langfelder and Horvath, 2008), using all expressed genes that had a fragments per kilobase of transcript per million mapped reads (FPKM) ≥ 5 for at least one sampling time point x temperature combination. Scale-free co-expression network analysis was conducted with a minModuleSize of 30 and a soft threshold of 7, which was determined from analysis of scale-free topology. A dynamic tree cutoff of 0.20 was used to combine similar trees. Eigengenes were computed for each module and correlated to root respiration rate using Spearman’s rank correlation. Hubs for gene modules were identified using the “chooseTopHubInEachModule” function in the WGCNA package. For all analyses p ≤ 0.05.

## 3 Results

### 3.1 Storage effects on root transcriptome

**Figure 1:**
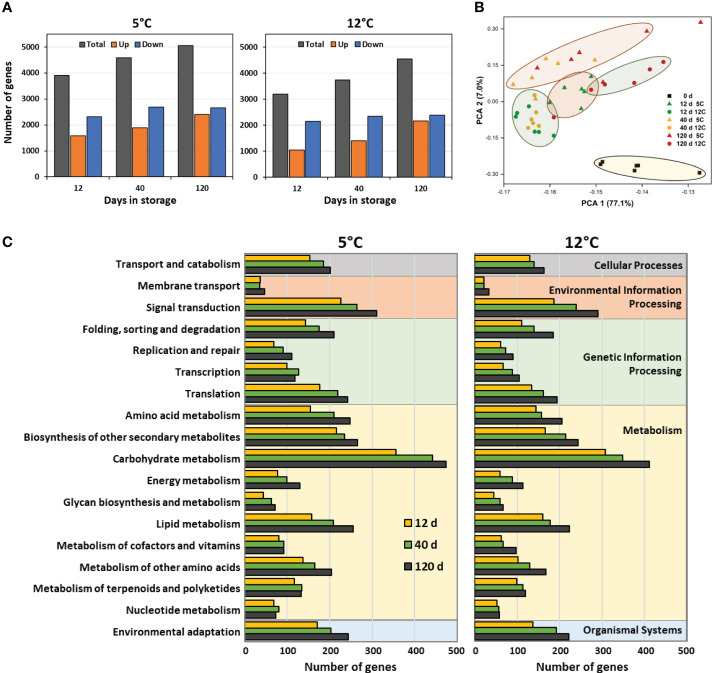
Effects of storage duration and storage temperature on the sugarbeet root transcriptome for roots stored for 12, 40 or 120 d at 5 or 12°C. **(A)** Total number of differently expressed genes (DEGs), relative to the day of harvest, and number of upregulated and downregulated DEGs, as a function of storage duration and temperature. Some genes are differentially expressed at one or more time point or temperature; therefore, summation of data in figure does not equal the total number of genes that were differentially expressed in storage. **(B)** Diversity between transcriptomes of roots at harvest and after 12, 40 or 120 d storage at 5 or 12°C as defined by principal component analysis (PCA) of all expressed genes. **(C)** Functional diversity of DEGs displayed as the population of DEGs within roots stored for 12, 40, or 120 d at 5 or 12°C that assign to the 18 most highly populated KEGG functional orthologies.

RNA sequencing identified 25,637 expressed genes in postharvest sugarbeet roots. Of these genes, 8656 genes, or 34% of the total, were differentially expressed during 120 d storage at 5 or 12°C. Alterations in the sugarbeet root transcriptome were evident within two weeks after harvest, with approximately 3900 and 3200 genes differentially expressed in roots stored at 5 and 12°C, respectively, after 12 d storage (**Figure 1A**). Transcriptomic changes increased with time in storage, and by 120 d approximately 5100 and 4500 genes were differentially expressed in roots stored at 5 and 12°C, respectively. Both up-regulated and down-regulated genes were found in stored roots. Down-regulated genes, however, were more prevalent than up-regulated genes throughout storage regardless of storage temperature. While differentially expressed genes (DEGs) were abundant at both storage temperatures, DEGs were 16% more abundant, on average, in roots stored at 5°C than in roots stored at 12°C. Gene identifiers, expression data, and annotations for all DEGs and gene identifier and expression data for all expressed genes are available in **Supplementary Tables S1A, B**.

Principal component analysis (PCA) of expressed genes in freshly harvested and stored roots confirmed that storage was responsible for significant changes to the root transcriptome (**Figure 1B**). This was evidenced by the distinct separation of roots at harvest from all stored root samples in the PCA. Among stored roots, those at 5°C were generally separate from those at 12°C, indicating that storage temperature also altered the root transcriptome. At 5°C, roots stored for 12 d separated in the PCA from those stored for 40 and 120 d, while at 12°C, overall transcriptomic changes were similar for roots stored for 12 and 40 d, but distinctly different from roots stored for 120 d. Storage duration, therefore, affected changes to the transcriptome, although the effect of storage duration differed between the two temperatures.

Storage-related DEGs contributed to a diverse range of cellular and molecular functions as revealed by functional classification of DEGs using KEGG identifiers (**Figure 1C**). Carbohydrate metabolism was the functional classification to which the greatest number of storage-related DEGs mapped, irrespective of storage temperature or duration. Other functional classifications that were highly populated with DEGs included, in descending order, signal transduction, biosynthesis of secondary metabolites, lipid metabolism, amino acid metabolism, and environmental adaptation. Cellular functions and relative abundance of DEGs per functional class were similar for roots stored at 5 or 12°C indicating that functional changes in gene expression were similar at both storage temperatures. Gene identifiers for DEGs contributing to the functional classifications of **Figure 1C** are available in **Supplementary Table S2**.

**Figure 2:**
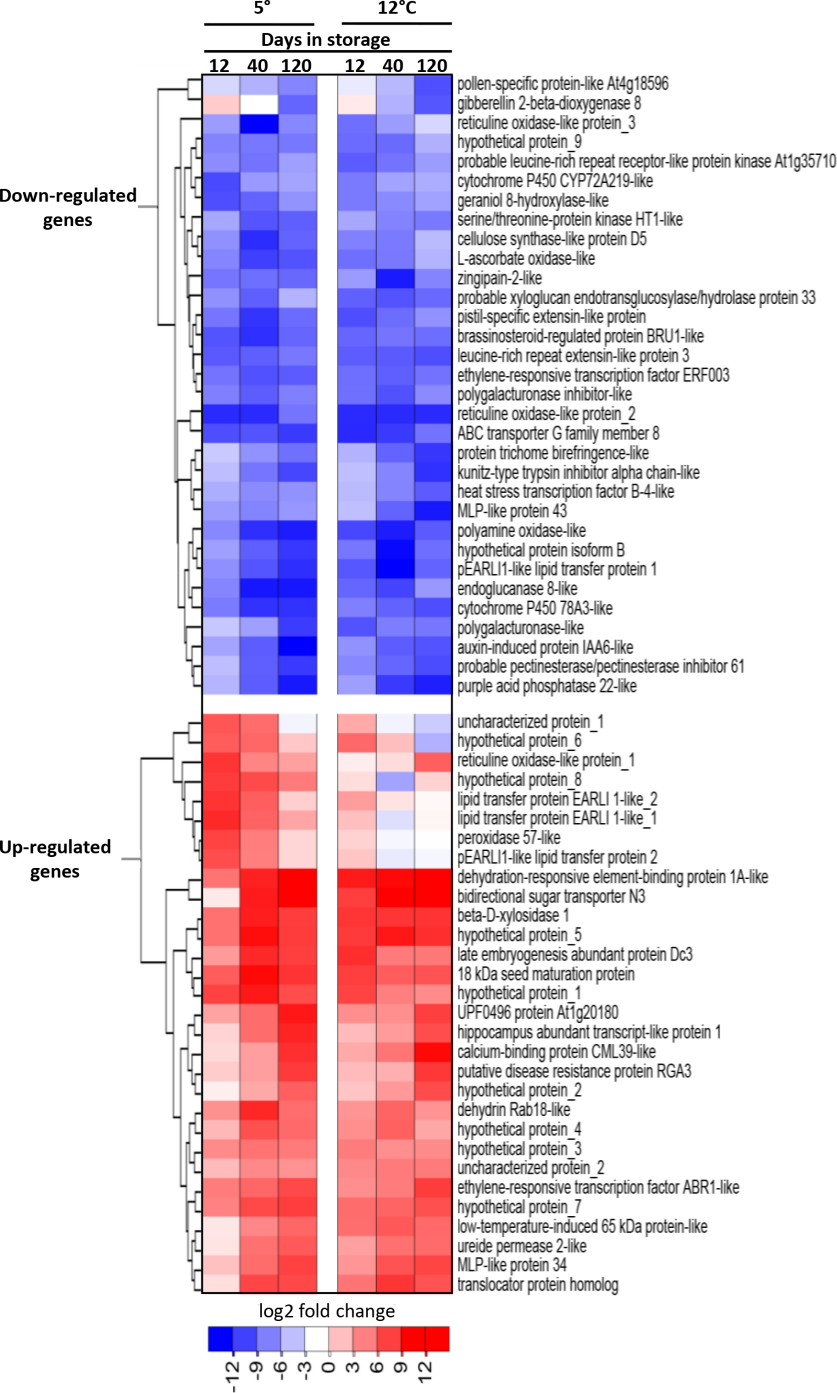
Heat map of the changes in expression with respect to storage duration and storage temperature for the most highly upregulated and downregulated genes in stored sugarbeet roots. Genes are the ten most upregulated and ten most downregulated genes for each storage duration x storage temperature combination, with genes hierarchically clustered based on expression similarities. Data are the log_2_ fold change in gene expression relative to expression on the day of harvest.

Genes that were highly up-regulated and down-regulated in storage were identified by compiling the ten most up-regulated and the ten most down-regulated genes at each sampling time (12, 40 and 120 d) for roots stored at 5 and 12°C (**Figure 2**). Highly up-regulated genes included many genes for uncharacterized proteins (10 of 30 genes) as well as a gene for a bidirectional sugar transporter, four genes involved in plant responses to cold and dehydration, and three lipid transferase genes. Within the 32 most down-regulated genes were five genes involved in hormone metabolism and signaling, five oxidase genes, and five genes contributing to cell wall metabolism. Expression data and gene identifiers for all highly up-regulated and down-regulated genes is available (**Supplementary Table S3**).

### 3.2 Storage effects on root metabolome

**Figure 3:**
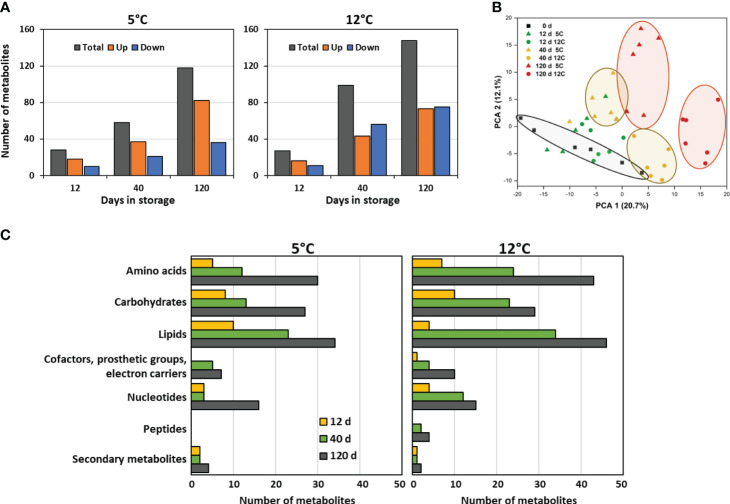
Effects of storage duration and storage temperature on the sugarbeet root metabolome for roots stored for 12, 40, or 120 d at 5 or 12°C. **(A)** Total number of metabolites that were significantly altered in concentration, relative to the day of harvest, and number of metabolites that significantly increased or decreased in concentration, as a function of storage duration and temperature. Some metabolites are altered in concentration at one or more time point or temperature; therefore, summation of data in figure does not equal the total number of metabolites that changed in concentration during storage. **(B)** Diversity between metabolomes of roots at harvest and after 12, 40 or 120 d storage at 5 or 12°C as defined by principal component analysis (PCA) of all detected metabolites. **(C)** Functional classification of metabolites that were altered in concentration relative to the day or harvest displayed as the population of these metabolites within roots stored for 12, 40, or 120 d at 5 or 12°C that belong to each metabolite class.

HPLC-MS/MS identified and quantified 379 metabolites in postharvest roots. Of these metabolites, 225 metabolites, or 59% of the total, were significantly altered in concentration during 120 d storage at 5 or 12°C (**Figure 3A**). Statistically significant changes in metabolite concentrations were few after 12 d in storage, with fewer than 30 compounds altered at either storage temperature. However, with increased time in storage, the number of metabolites that were significantly altered in concentration increased by more than 4- to 5-fold, and by 120 d in storage, 118 and 148 compounds had changed in concentration at 5 and 12°C, respectively. Metabolomic changes increased with storage temperature, and after 120 d in storage, 25% more metabolites were altered in concentration in roots stored at 12°C than in roots at 5°C. A list of the metabolites that were significantly altered in concentration during storage, their functional classifications, and their relative concentrations at harvest and throughout storage is available in **Supplementary Table S4**.

Principal component analysis of the metabolic profiles of freshly harvested and stored roots confirmed that both storage duration and storage temperature affected the root metabolome (**Figure 3B**). Roots stored for 40 and 120 d were distinctly segregated in the PCA from roots on the day of harvest, with segregation from the freshly harvested roots increasing with time in storage. Dissimilarity in metabolomes, therefore, increased with time in storage. Temperature also clearly affected the root metabolome, as roots stored at 5°C clearly segregated from those stored at 12°C after 40 or 120 d storage.

Functional classification of the metabolites that were altered in concentration during storage indicated that storage-related changes to the metabolome were diverse (**Figure 3C**). Lipids were the most abundantly altered compounds during storage. Also abundantly altered were amino acids and carbohydrates. Changes in metabolites by functional classification were similar in both type and relative abundance for roots stored at 5 or 12°C.

**Figure 4:**
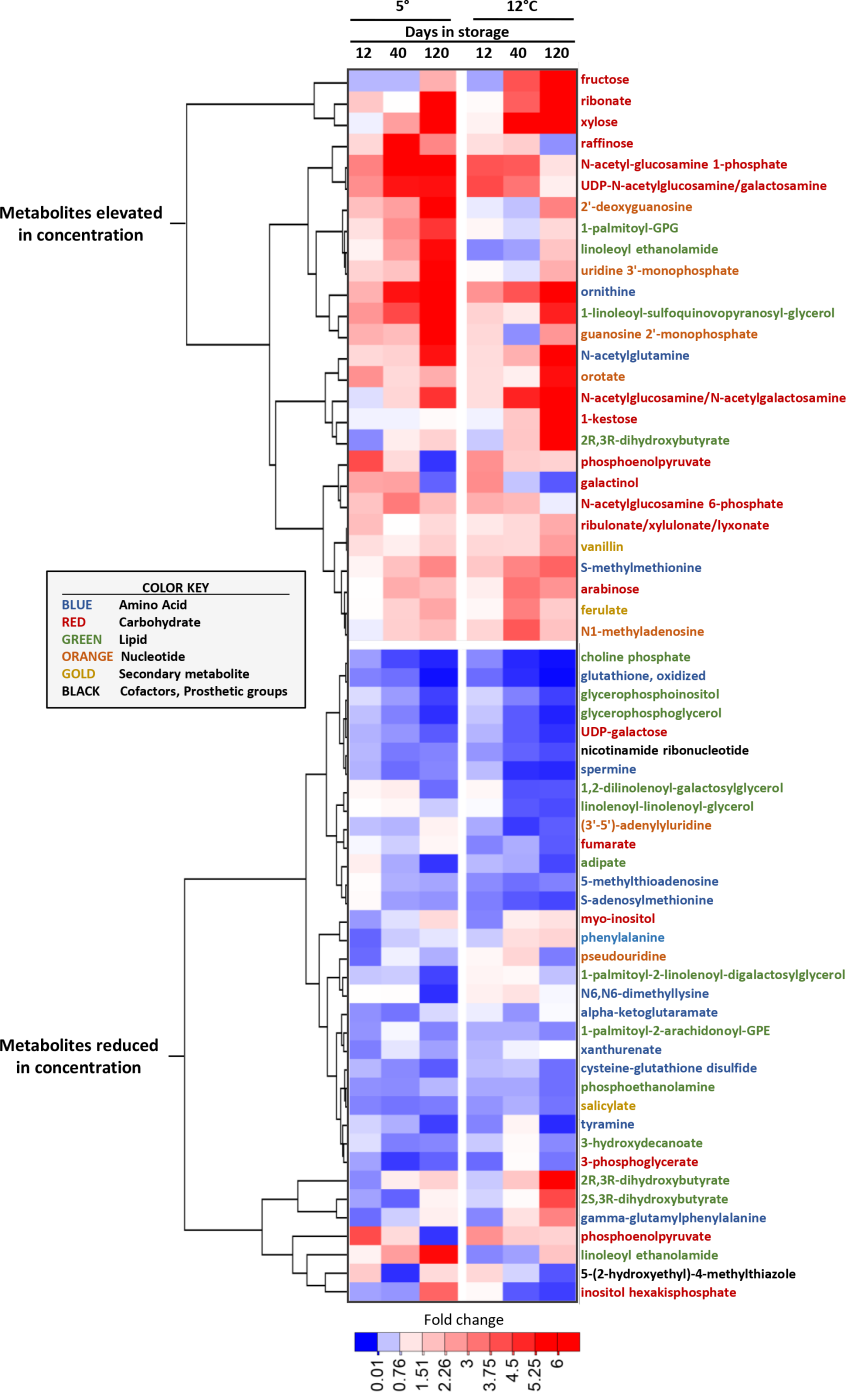
Heat map of the changes in metabolite concentration with respect to storage duration and storage temperature for the metabolites that were most increased and decreased in concentration in stored sugarbeet roots. Metabolites are the ten most elevated and the ten most reduced in concentration for each storage duration x storage temperature combination, with metabolites hierarchically clustered based on similarities in their concentration profiles. Data are the fold change in concentration relative to a metabolite’s concentration on the day of harvest. Font color of metabolite names denotes a metabolite’s classification as per the color key presented in the figure.

Metabolites that were highly elevated or reduced, based on their fold-change in concentration during storage, were identified by compiling the ten most elevated and reduced metabolites at each sampling time for roots stored at 5 and 12°C (**Figure 4**). Nearly half of the metabolites that were highly elevated during storage were carbohydrates, with fructose, which increased in concentration by 25-fold in roots stored at 12°C for 120 d, being the most highly elevated metabolite during storage. Of metabolites that were highly reduced in concentration during storage, lipids and amino acids were abundantly represented, and made up 37 and 31%, respectively, of the total. Carbohydrates, however, were less common and comprised only 17% of the most highly reduced metabolites.

### 3.3 Storage effects on respiration

**Figure 5:**
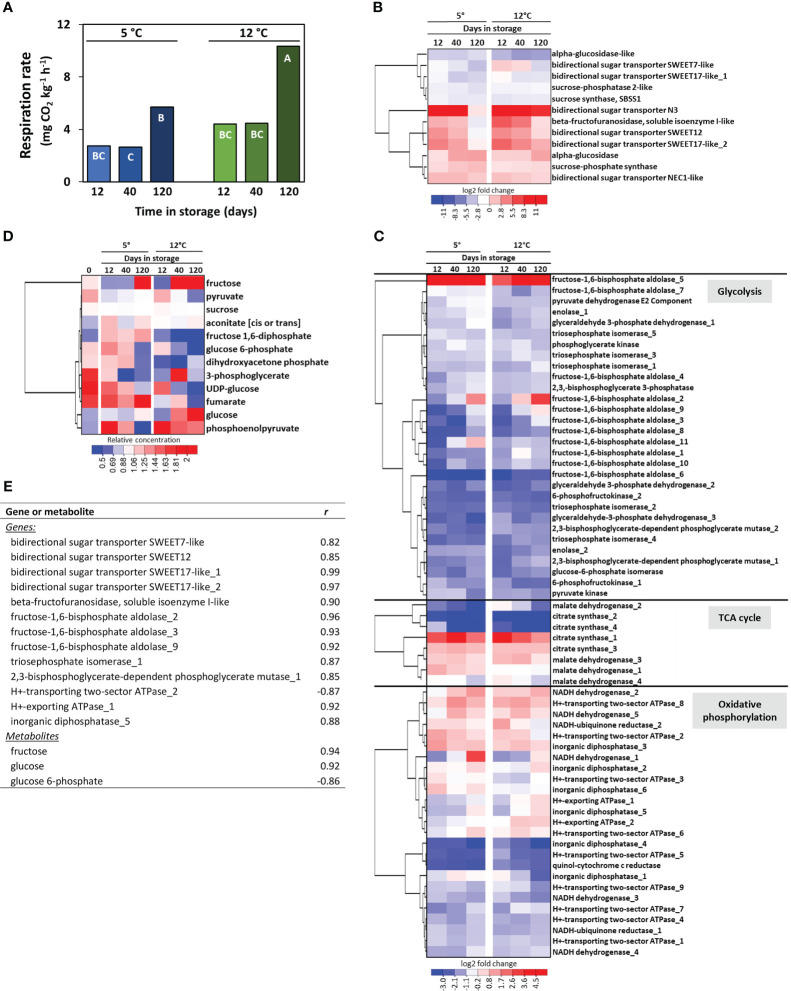
Effects of storage duration and storage temperature on sugarbeet root respiration rate and respiratory pathway gene expression and metabolite concentrations for roots stored for 12, 40 or 120 d at 5 or 12°C. **(A)** Root respiration rate as a function of storage duration and temperature. Bars labelled with different letters are significantly different based on Fisher’s LSD (p ≤ 0.05). **(B)** Heat map of the changes in gene expression for genes involved in sucrose transport and metabolism that were significantly altered in expression during storage, with genes hierarchically clustered based on similarities in expression. Data are the log_2_ fold change in expression relative to expression on the day of harvest. **(C)** Heat map of the changes in gene expression for genes involved in glycolysis, the tricarboxylic acid (TCA) cycle, and oxidative phosphorylation that were significantly altered in expression during storage, with genes hierarchically clustered based on similarities in expression. Data are the log_2_ fold change in expression relative to expression on the day of harvest. **(D)** Heat map of changes in respiratory pathway metabolites for those metabolites that were significantly altered in concentration during storage, with metabolites hierarchically clustered based on similarities in their concentration profiles. Data are the fold change in concentration relative to a metabolite’s concentration on the day of harvest. **(E)** Genes and metabolites in the respiratory pathway that were significantly altered in expression or concentration during storage and significantly correlated with root respiration rate, where *r* is the Pearson correlation coefficient. Gene identifiers for all genes included in the Figure are available (**Supplementary Table S5**).

Transcriptomic and metabolomic data were additionally examined to identify genes and metabolites that potentially participate, regulate, or serve as markers for sugarbeet root storage respiration rate. Root respiration rate was influenced by both storage temperature and storage duration, with respiration, on average, 70% greater at 12°C than at 5°C and elevated more than 2-fold after 120 d of storage relative to 12 and 40 d storage (**Figure 5A**). A total of 75 genes involved in the respiration of sucrose were differentially expressed at one or more time points during storage (**Figures 5B, C**). Twelve of these DEGs were involved in the remobilization of sucrose from storage and sucrose metabolism (**Figure 5B**) and included six genes for bidirectional sugar transporters that transport sugars across tonoplast and plasma membranes for the remobilization and intercellular movement of sucrose, and two genes (sucrose synthase 1 and β-fructofuranosidase) that catabolize sucrose to its constituent monosaccharides. Genes involved in sucrose remobilization and metabolism were predominantly upregulated during storage with one gene (bidirectional sugar transporter N3) up-regulated more than 52,000-fold. An additional 63 genes contributed to the respiration of glucose and fructose to carbon dioxide, water, and energy (**Figure 5C**). Of these genes, 30 encoded enzymes of the glycolytic pathway, eight encoded TCA cycle enzymes, and 25 participated in oxidative phosphorylation. Genes encoding eight of the ten reactions of glycolysis were differentially expressed during storage. These DEGs were predominantly down-regulated, although two fructose-1,6-bisphosphate aldolase (FBA) genes (FBA_2 and FBA_5) were up-regulated by as much as 14 and 160-fold, respectively. DEGs participating in the TCA cycle encoded only for the first (citrate synthase) and last (malate dehydrogenase) enzymatic steps in the pathway. Several of these DEGs were strongly down-regulated during storage while only a single gene (citrate synthase_1) was highly up-regulated. DEGs within the oxidative phosphorylation pathway catalyzed six reactions in the electron transport chain and ATP biosynthesis. Five of the 25 DEGs in the oxidative phosphorylation pathway encoded for electron transport chain complex 1 (NADH dehydrogenase), while 17 genes were involved in ATP synthesis.

Despite the abundance of respiratory pathway DEGs, only twelve respiratory pathway metabolites were significantly altered in concentration during storage (**Figure 5D**). These included sucrose, the immediate products of sucrose catabolism (glucose, fructose, and UDP-glucose), six glycolytic intermediates, and two intermediates of the TCA cycle. Metabolites that increased in concentration in roots stored at 5 and 12°C included glucose, fructose, and aconitate, while fructose 1,6-diphosphate increased only in roots stored at 5°C and phosphoenolpyruvate increased only in roots stored at 12°C. Of the six glycolytic intermediates that were altered during storage, all declined in concentration except for fructose 1,6-diphosphate at 5°C and phosphoenolpyruvate at 12°C. Sucrose, the initial substrate for respiration, declined by an average of 4.3% during 120 d storage.

Correlation analysis of root respiration rate with respiratory pathway DEGs and metabolites identified twelve DEGs that positively correlated with root respiration rate and one DEG that negatively correlated with respiration rate (**Figure 5E**). Among positively correlated DEGs were genes for four bidirectional sugar transporters, a β-fructofuranosidase, five glycolytic enzymes, and two oxidative pathway proteins, while a gene involved in oxidative phosphorylation was negatively correlated to respiration rate. No TCA cycle DEGs were significantly correlated with root respiration rate. Two genes encoding bidirectional sugar transporter SWEET17 were the most highly correlated in their expression to root respiration rate. Three respiratory pathway metabolites also correlated significantly with respiration rate, with glucose and fructose positively correlated and glucose 6-phosphate negatively correlated to root respiration rate.

**Figure 6:**
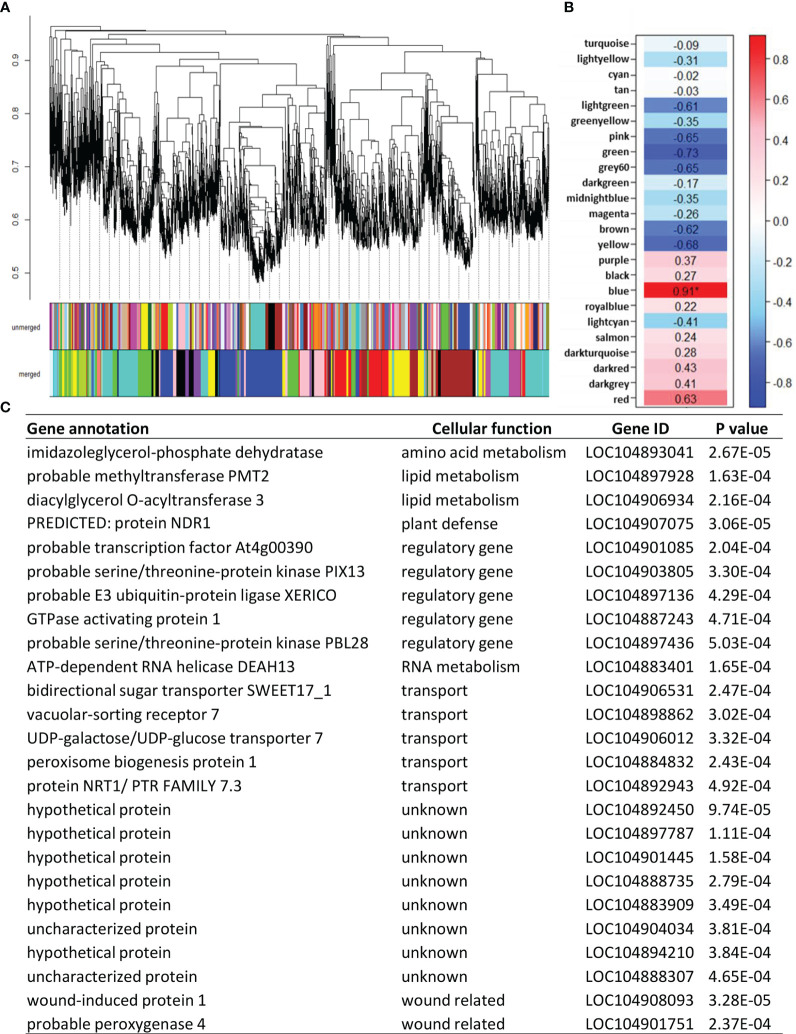
Correlation of all appreciably expressed genes in postharvest sugarbeet roots to root respiration rate using weighted gene co-expression network analysis (WGCNA). **(A)** Hierarchal clustering of expressed genes and assignment of co-expressed genes to color-coded modules before (unmerged) and after (merged) combining modules with ≥ 80% similarity. **(B)** Correlation of root respiration rate with eigengenes for each merged, color-coded module. Values within heat map are correlation coefficients, with significant correlation denoted by an asterisk. **(C)** Twenty-five most highly correlated genes from the ‘blue’ co-expression module that was significantly correlated to respiration rate and their gene identifiers, cellular function, and correlation p-values.

Additional genes that potentially regulate or serve as markers for sugarbeet root storage respiration rate were identified using weighted gene co-expression network analysis (WGCNA). Unlike the correlation analysis described above that utilized only those genes that were differentially expressed and directly involved in the sucrose respiratory pathway, WGCNA evaluated root respiration rate against all genes that were appreciably expressed in postharvest sugarbeet roots. WGCNA categorized 13,354 postharvest-expressed genes into 25 color-coded modules based on similarities in their postharvest expression after combination of modules with 80% or more similarity (**Figure 6A**). Correlation of eigengenes for each module with root respiration rate identified a statistically significant, positive relationship between respiration rate and genes in the blue module (**Figure 6B**). The blue module was comprised of 1906 genes (**Supplementary Table S6**). Of these genes, the 25 genes that were most highly correlated to respiration rate are presented (**Figure 6C**). Bidirectional sugar transporter SWEET17_1 was again identified for its high correlation to root respiration rate. Also highly correlated to root respiration rate were an additional four genes involved in transport, five regulatory genes and eight uncharacterized genes.

**Figure 7:**
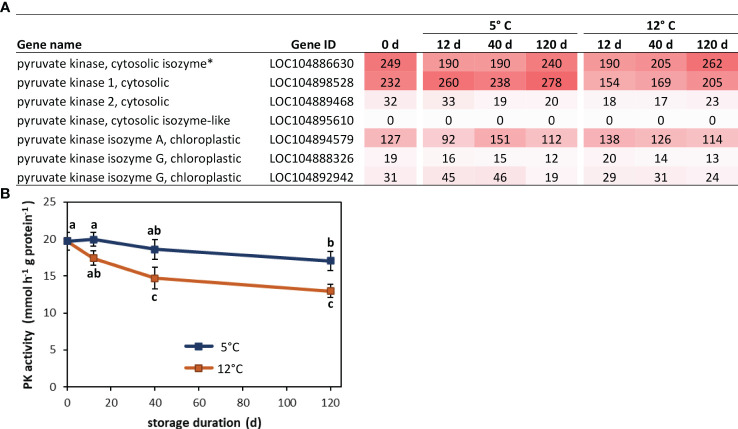
Pyruvate kinase (PK) gene expression and enzyme activity in postharvest sugarbeet roots. **(A)** Fragments per kilobase of transcript per million mapped reads (FPKM) values from RNA sequencing for all sugarbeet PK isoforms at harvest and after 12, 40 or 120 d storage at 5 or 12°C. PK isoform identified as hub for genes positively correlated with root respiration rate by weighted gene co-expression network analysis (WGCNA) is denoted with an asterisk. **(B)** PK enzyme activity at harvest and during 120 d storage at 5 or 12°C. Data points with different letters are significantly different based on Fisher’s LSD (p ≤ 0.05).

Analysis of functional associations between blue module genes identified a cytosolic pyruvate kinase (PK) as the hub gene for the module. As the module hub, this PK gene was the most central and most highly interconnected gene in a network analysis of module genes based on their biological functions (Chen et al., 2017). The identified hub gene (LOC104886630) is one of seven pyruvate kinases found in sugarbeets, one of six PK genes expressed in postharvest roots, and one of two highly expressed cytosolic pyruvate kinase genes in stored sugarbeet roots (**Figure 7A**). Expression of this gene declined transiently during storage, with a 23-24% reduction in expression within 12 d of storage and a return to harvest levels of expression after prolonged storage. Although both storage temperatures exhibited similar changes in expression as a function of storage duration, expression rebounded more rapidly and robustly in roots stored at 12°C relative to those stored at 5°C. In contrast, PK enzymatic activity declined by 14 and 34% after 120 d storage at 5 and 12°C, respectively (**Figure 7B**).

## 4 Discussion

An analysis of the transcriptomic and metabolomic changes that occur during postharvest storage of sugarbeet roots documents the massive and diverse changes in metabolism that roots undergo following harvest. A total of 8656 genes, or 34% of all expressed genes, changed in expression in roots stored for up to 120 d at 5 or 12°C, relative to expression at harvest. Similarly, 225 metabolites, or 59% of the total number of metabolites detected in harvested roots, significantly changed in concentration relative to their concentrations at harvest. Genes that were differentially expressed during storage participated in a wide range of metabolic functions, including primary carbon and nitrogen metabolism and secondary metabolic pathways, and contributed to diverse cellular functions including transcription, translation, replication, transport, signaling, and environmental adaptation. Similarly, metabolites that were altered in concentration during storage belonged to diverse classes of compounds, including carbohydrates, lipids, amino acids, secondary metabolites, nucleotides, cofactors, and electron carriers. These transcriptomic and metabolomic changes are assumed to be manifestations of endogenous sugarbeet root responses to storage temperature and duration, since roots exhibited no visible symptoms of disease even after prolonged storage at 12°C. Although storage diseases, especially fungal rots, can develop after harvest (Campbell and Klotz, 2006; Madritsch et al., 2020), disease development was avoided in this study by obtaining roots from disease-free greenhouse plants that were grown in a soilless peat mix, carefully harvested by hand to minimize injuries, and washed free of all potting media prior to storage in controlled environment chambers. Moreover, annotation of RNA sequencing data against the sugarbeet genome (Dohm et al., 2013) further guaranteed that only sugarbeet genes were included in the analysis of data.

The extensive and diverse changes in the transcriptome and metabolome document a major reorganization in metabolism and cellular functioning in sugarbeet roots after harvest. Such a reorganization is perhaps not surprising due to the physiological transitions and environmental stresses encountered by harvested roots. Upon harvest, sugarbeet taproots are removed from their sources of photosynthate, water, and nutrients and forced to transition from a growing, sucrose-importing sink organ to a non-growing organ that must remobilize stored sucrose for its metabolism. Harvest and piling operations additionally inflict cuts, scrapes, bruises, and/or cracks to roots with such frequency that all roots are injured prior to storage (Steensen, 1996; Wiltshire and Cobb, 2000) and must activate wound-healing processes to limit dehydration and protect against pathogenic organisms (Fugate et al., 2016). Cold storage temperatures used to retard growth of storage pathogens and slow root respiration subject roots to cold stress and fulfill the biennial root’s vernalization requirement, initiating the transition from a vegetative to a reproductive lifecycle (Rodrigues et al., 2020). While pile ventilation facilitates the cooling of piles, it also dehydrates roots, adding further stress to stored roots (Wyse, 1973; Van Eerd et al., 2012).

Both storage duration and storage temperature significantly impacted root transcriptional and metabolomic changes during storage. Overall, the frequency of gene expression and metabolite concentration changes increased with time in storage. Changes in the transcriptome were rapid, with the expression of more than 3000 genes altered after only 12 d in storage, while metabolite concentration changes were notably slower, with fewer than 30 metabolites altered in concentration after 12 d storage. Regardless of storage duration, more genes were differentially expressed at 5°C than 12°C, a likely reflection of the additional stress and the satisfaction of vernalization requirements by the colder storage temperature (Milford, 2006). In contrast, more metabolites were altered in concentration at 12°C than at 5°C, a likely response to the greater metabolic activity in roots stored at the higher temperature (Kays and Paull, 2004).

Because sucrose loss during storage is largely caused by root respiration (Wyse and Dexter, 1971b; Vukov and Hangyál, 1985), an analysis of the genes and metabolites that participate in the respiration of sucrose was carried out to identify genes that participate in and potentially regulate and/or serve as biomarkers for this pathway. In this study, sugarbeet roots lost an average of 4.3% of the sucrose present at harvest during 120 d storage, a significant decline since sucrose comprises 75-80% of root dry matter (Bohn et al., 1998). Like other postharvest plant products, sugarbeet root respiration rate increased with an elevation in storage temperature (Kays and Paull, 2004). Prolonged storage also increased respiration rate in this study as well as in earlier storage studies (Wyse, 1978; Fugate et al., 2023b), although the cause for this respiratory increase is unknown. A total of 75 respiratory pathway genes and 12 respiratory pathway metabolites that participate in the remobilization or degradation of sucrose, glycolysis, the TCA cycle, or oxidative phosphorylation were altered in expression or concentration at some time during 120 d storage at 5 and 12°C. Of these 87 genes and metabolites, 12 genes and two metabolites significantly and positively correlated in expression or concentration with root respiration rate, including four genes for bidirectional sugar transporters, a gene for β-fructofuranosidase, five glycolytic enzyme genes, two genes for oxidative pathway proteins and the metabolites, fructose and glucose. No TCA cycle genes, however, correlated to root respiration rate, indicating that this pathway is unlikely to have a role in restricting root respiration, a conclusion that was also reached in an earlier study that examined enzymatic changes during storage (Megguer et al., 2017).

The genes most highly correlated to root respiration rate were two genes encoding isoforms of bidirectional sugar transporter SWEET17. SWEET (Sugars Will Eventually be Exported Transporter) proteins are membrane-localized transporters that facilitate nondirectional passage of carbohydrates across cellular membranes (Jeena et al., 2019). In most plant species, SWEET17 localizes to the tonoplast, and in *Arabidopsis*, this gene specifically transports fructose (Chardon et al., 2013; Guo et al., 2014; Zhou et al., 2018; Liu et al., 2019; Zhang et al., 2021). While localization to the tonoplast is probable for sugarbeet SWEET17 genes based on sequence homology to other tonoplast-localized SWEET genes, specificity for fructose transport in sugarbeet cannot be presumed since substrate specificities for SWEET proteins vary between plant species (Kryukov et al., 2021). Genes for two other bidirectional sugar transporters, SWEET7 and SWEET12, also positively correlated to respiration rate. These transporters likely facilitate sugar transport across the plasma membrane based on homology to characterized genes in other plant species (Chen et al., 2012; Zhang et al., 2021). Since sugarbeet roots sequester stored sucrose in vacuoles, tonoplast-localized transporters are likely to be important in remobilizing stored sucrose into the cytoplasm where it is metabolized to generate respiratory substrates (Hedrich et al., 2015). A role for plasma membrane-localized sugar transporters in root respiration is less obvious, although the high correlation between these transporters and root respiration rate hints that intercellular movement of sugars is also involved in providing respiratory substrate to respiring cells.

Five enzymes of the glycolytic pathway correlated to root respiration rate, including three isozymes of fructose-1,6-bisphosphate aldolase, a triosephosphate isomerase, and a 2,3-bisphosphoglycerate-dependent isozyme of phosphoglycerate mutase (PGlyM). Fructose-1,6-bisphosphate aldolase, commonly referred to as aldolase (ALD), catalyzes the cleavage of fructose-1,6-bisphosphate into two triosephosphates. Although three genes encoding this enzyme correlated to respiration rate, ALD is not classically regarded as having a regulatory function in glycolysis or respiration (Plaxton, 1996; Plaxton and Podestá, 2006). Similarly, triosephosphate isomerase (TPI) which catalyzes interconversion of the triosephosphates, dihydroxyacetone phosphate and glyceraldehyde 3-phosphate, is not typically regarded as a regulatory enzyme for glycolysis or respiration, although Madritsch et al. (2020) found a gene encoding a different TPI isozyme to have a central role among genes that were correlated with sucrose loss in stored sugarbeet roots. The phosphoglycerate-dependent isozyme of phosphoglycerate mutase (PGlyM) that correlated to respiration rate is also unlikely to have a major role in regulating glycolysis or respiration since PGlyM activity in plants is primarily due to isozymes that are not dependent on 2,3-bisphosphoglycerate as a cofactor (Duminil et al., 2021).

A β-fructofuranosidase gene encoding a soluble acid invertase, as well as the metabolic reaction products of invertase activity (i.e., fructose and glucose), also correlated strongly with root respiration rate. Two previous studies have reported correlation of acid invertase activity with sucrose loss in stored sugarbeet roots (Wyse, 1974; Berghall et al., 1997). However, in these earlier studies, it was unclear whether invertase activity originated from sugarbeet roots or from pathogenic organisms present on the stored roots (Klotz and Finger, 2004). In the current study, β-fructofuranosidase gene expression increased with storage duration and temperature. Expression levels for this gene, however, were at negligible levels from harvest through 120 d storage at both storage temperatures (**Supplementary Table S1**) raising questions of the importance of this gene in respiratory sucrose loss. Similarly, soluble acid invertase enzyme activity was previously reported to increase during storage, but was only present at barely detectable activities at harvest and throughout storage in roots exhibiting no disease symptoms (Klotz and Finger, 2004).

An additional 1896 genes that positively correlated to respiration rate were identified by weighted gene co-expression network analysis. Since the analysis utilized expression data for all appreciably expressed genes in postharvest sugarbeet roots, WGCNA identified not only genes that directly participate in the respiratory pathway, but also regulatory, signaling and transporter genes, and genes of unknown function. Of particular note among the 25 genes most highly correlated with respiration rate in this analysis were five regulatory genes, five transporter genes, and eight genes that encode proteins of unknown function. Highly correlated regulatory genes included transcription factors, protein kinases and other signaling compounds that may be involved in governing sugarbeet root postharvest respiration. Highly correlated transport genes included a previously described SWEET17 gene, that putatively transports sugars between the vacuole and cytoplasm, and a vacuolar-sorting receptor that is likely involved in protein import into the vacuole (Miao et al., 2006). Other transporters that were highly correlated to respiration rate are involved in lipid transport into peroxisome membranes (peroxisome biogenesis protein 1; Lopez-Huertas et al., 2000), transfer of nucleotide sugars into the endoplasmic reticulum (UDP-galactose/UDP-glucose transporter 7; Reyes et al., 2006) and auxin transport between cells (protein NRT1/PTR family 7.3; Watanabe et al., 2020). Also high correlated with respiration rate were an abundance of genes of unknown function. These uncharacterized genes are a fitting reminder of our limited knowledge of the metabolic and physiological changes that occur in postharvest sugarbeet roots and the genes and proteins that effect and control these changes.

Network analysis of protein-protein interactions identified a pyruvate kinase gene as the central hub for the nearly 2000 genes that correlated to root respiration rate in WGCNA. Hubs are genes that are highly interconnected to other genes within co-expression modules and often function as regulators or biomarkers for module-correlated phenotypic traits (Chen et al., 2017; Wang et al., 2022). Pyruvate kinase catalyzes the conversion of phospho*enol*pyruvate to pyruvate in the final enzymatic reaction in the glycolytic pathway. Identification of PK as a possible regulator of storage respiration rate agrees with earlier results that assigned control of sugarbeet root respiration rate to the glycolytic pathway and identified PK as a prime regulator of this pathway (Megguer et al., 2017). In fact, in most plant species, PK activity is considered to be the primary controller of glycolysis (Givan, 1999; Plaxton and Podestá, 2006). The PK gene identified as the hub for respiration-correlated genes encodes one of two highly expressed, cytoplasmic isozymes in postharvest sugarbeet roots. Expression of this gene differed from PK enzymatic activity during storage, but this was perhaps not surprising since PK enzymatic activity derives from the combined expression of six PK genes in postharvest sugarbeet roots and post-transcriptional and post-translational regulation of pyruvate kinases are known to occur in other organisms (Vaulont et al., 1986; Plaxton, 1996).

## 5 Conclusions

The extensive transcriptional and metabolomic changes observed in postharvest sugarbeet roots provides clear evidence of the massive and diverse physiological and metabolic changes that occur in stored roots. These changes likely are needed for roots to transition at harvest from a sucrose accumulating organ to a sucrose utilizing organ, initiate wound-healing activities in response to harvest and piling injuries, adapt to cold temperature and dehydration stresses inflicted on roots by the storage environment, and transition from a vegetative to a reproductive lifecycle as roots are vernalized in storage. Storage temperature and duration further impacted root transcriptional and metabolomic changes. Alterations in gene expression and metabolite concentrations increased with time in storage, while storage at 5 and 12°C increased the frequency of transcriptional changes and metabolomic changes, respectively. Numerous genes correlated in expression with root respiration, the primary cause of postharvest sucrose loss. Most notable among these genes were two isoforms of a bidirectional sugar transporter, SWEET17, that may have a role in remobilizing sucrose from the storage vacuole into active metabolism in the cell cytoplasm, and a pyruvate kinase gene that is potentially involved in the regulation of glycolysis and respiration rate in stored sugarbeet roots. The usefulness of these genes as biomarkers for sugarbeet root storage respiration rate remains to be determined. However, the establishment of any of these genes as biomarkers for postharvest respiration rate would provide a useful tool for selecting sugarbeet germplasm for reduced respiratory sucrose loss during storage.

## Data availability statement

The datasets presented in this study can be found in online repositories. The names of the repository/repositories and accession number(s) can be found below: MetaboLights accession number MTBLS1106. National Center for Biotechnology Information (NCBI) Sequence Read Archive (SRA) accession number PRJNA938134.

## Author contributions

KF: Conceptualization, Formal analysis, Writing – original draft. JE: Data curation, Formal analysis, Investigation, Writing – review & editing. AL: Formal analysis, Investigation, Writing – review & editing. MT: Formal analysis, Writing – review & editing. CC: Supervision, Writing – review & editing. MK: Supervision, Writing – review & editing. FF: Formal analysis, Investigation, Writing – review & editing.
